# Platelet-Derived Growth Factor Receptor Alpha as a Marker of Mesenchymal Stem Cells in Development and Stem Cell Biology

**DOI:** 10.1155/2015/362753

**Published:** 2015-07-16

**Authors:** Ramin M. Farahani, Munira Xaymardan

**Affiliations:** ^1^Institute of Dental Research, Department of Life Science, Faculty of Dentistry, University of Sydney, Sydney, NSW 2006, Australia; ^2^Bioengineering Unit, Department of Life Science, Faculty of Dentistry, University of Sydney, Sydney, NSW 2006, Australia

## Abstract

Three decades on, the mesenchymal stem cells (MSCs) have been intensively researched on the bench top and used clinically. However, ambiguity still exists in regard to their anatomical locations, identities, functions, and extent of their differentiative abilities. One of the major impediments in the quest of the MSC research has been lack of appropriate *in vivo* markers. In recent years, this obstacle has been resolved to some degree as PDGFR*α* emerges as an important mesenchymal stem cell marker. Accumulating lines of evidence are showing that the PDGFR*α* ^+^ cells reside in the perivascular locations of many adult interstitium and fulfil the classic concepts of MSCs *in vitro* and *in vivo*. PDGFR*α* has long been recognised for its roles in the mesoderm formation and connective tissue development during the embryogenesis. Current review describes the lines of evidence regarding the role of PDGFR*α* in morphogenesis and differentiation and its implications for MSC biology.

## 1. PDGFR***α*** and Mesenchymal Stem Cells

Adult organs/tissues are composed of both parenchymal cells and stromal interstitial cells. The latter provide structural support, paracrine signals, extracellular matrix, and a supply of progenitor cells in healthy growth and contribute to repair of the tissue in response to routine, minor damage. For example, the bone marrow (BM) is accepted to contain a stromal tissue niche of myelosupportive reticular cells that can also give rise to bone and fat [1]. In this way, the stromal elements play a key role in supporting parenchymal stem cell activity in haematopoiesis, while retaining inherent stem cell properties to allow coordinated growth of organ and tissue. A subfraction of the stromal cells form Colony-Forming-Fibroblast-Units (CFU-F), which are plastic adherent cells that can be differentiated into bone, cartilage, and fat* in vitro* [2]. Similar cells have been isolated from virtually all tissues that are denominated as the MSCs. The proposed regenerative and immunosuppressive properties of MSCs have led to numerous clinical trials exploring their utilities for the treatment of a variety of diseases including immunological and cardiovascular conditions, perhaps overzealously offered as panacea to the patient despite lack of understanding of these cells in their basic properties and functions* in vivo*.

MSCs are generally selected for their plastic adherent property due to lack of uniformed* in vivo* markers. Some of the markers described for their* in vitro* derivatives include CD105, CD166, CD90, CD44, CD29, CD73, CD9, CD13, and CD106, which do not directly correlate with their* in vivo* founders [3]. The lack of appropriate markers means that present definitions of MSCs emphasize generic properties of these cells and fails to distinguish subsets of stromal cells with specialized niche functions, inability to functionally dissect the important differences within the extended stromal cell family; for example, ambiguous distinctions between fibroblasts and MSCs, as well as MSCs with other progenitors (e.g., muscle satellite cells), may lead to contamination of the cultured MSCs, either compromising their function or generating false hope for the scope of their differentiation abilities. Moreover, due to the absence of* in vivo* marker labelling, little is known about their* in vivo* anatomical locations, immunophenotypes, developmental origin, and contribution to organogenesis as well as their roles in postnatal tissue homeostasis and injury.

**Figure 1:**
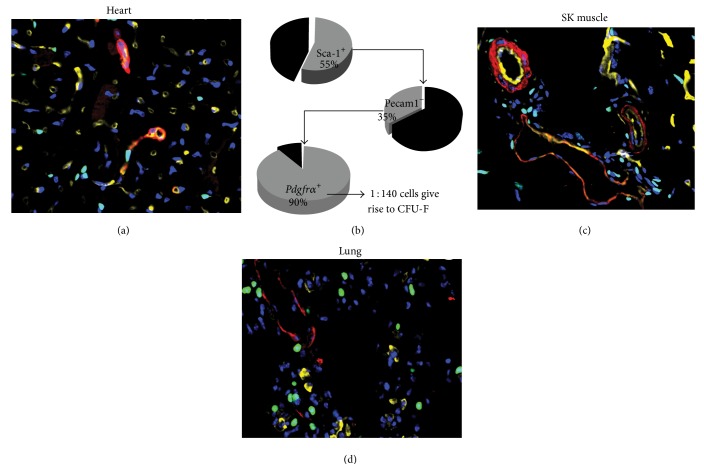
(a) Immunohistochemistry staining of smooth muscle alpha actin (red) and bandeiraea simplicifolia lectin (yellow) on tissues collected from* Pdgfrα*-GFP mouse (B6.129S4-Pdgfr*α* ^tm11(EGFP)Sor/J^). The green fluorescence represents the* Pdgfrα* positive cells. The image shows a broad expression of the cells in the heart (a) and CFU-F enrichment strategy for the cardiac SCA-1^+^ *Pdgfrα*-GFP^+^ cells (b). Similar localization Pdgfr*α*-GFP is seen in skeletal muscle and lung interstitium (c, d).

A promisingly emerging marker, the Platelet-Derived Growth Factor Receptor Alpha (PDGFR*α*), has recently been used to isolate cells that fulfil the definition of MSCs from several murine and human tissues including BM [4, 5] and the heart (Figure 1(a)) [6, 7], which seems to provide an enriching factor to the MSC cultures. For instance, the CFU-F frequency of PDGFR*α* and SCA-1 expressing cells is approximately 120,000-fold higher than that of unfractionated BM mononuclear cells. Similar markers were also used to isolate cardiac CFU-F forming cells that have 6-fold enrichment compared to unfractionated interstitial cells (Figure 1(b)) [6]. This identification of the PDGFR*α* as the MSC marker has enabled the revelation of a perivascular anatomical location of the cells in BM, heart, and skeletal muscle [5, 6, 8]. The finding has also facilitated* in vivo* transplantation of the freshly isolated BM MSCs into irradiated mice. In these assays, the PDGFR*α* cells were able to differentiate into osteocytes and adipocytes in the recipient BM niches and when isolated from the new host, they are able to form CFU-F [4, 5].

PDGFR*α* is often indicated in the pathological events such as fibrosis, arthrosclerosis, and cancer, perhaps reflective of a complex mechanism of the MSC function during pathogenesis. However, due to their relative quiescence in homeostasis, the function of the cells* in vivo* is difficult to be elucidated. In this sense, learning from the innate embryonic differentiation process of cells may provide insight into the differentiation trajectory of these cell types. Interestingly, PDGFR*α* has long been identified as a mesenchymal progenitor marker during embryonic development, especially following gastrulation to play important roles in the mesoderm differentiation and direct organogenesis. In adults, the PDGFR*α* seems to be broadly expressed in the perivascular stromal interstitium of the tissues, with a subfraction of the PDGFR*α* cells presenting MSC characteristics [5, 6, 8, 9]. It is possible that the MSCs derive from early stages and reside in the adult tissue, become latent until being stimulated into cell cycle, and differentiate during disease or tissue growth. Presence of such cells are debated due to the possibility that the CFU-F formation of the stromal cells may also represent circumstantial or probability dictated events. Nevertheless, harnessing these cell types may be as formidable a task as challenges currently faced by many other stem cell related studies. Therefore, in-depth understanding of the PDGFR*α*, its role in the embryological process, and relationship of the receptor-ligand interactions are equally crucial to the prospective isolation and differentiation of MSCs in the context of tissue homeostasis, repair, and regeneration.

## 2. *Pdgfr* ***α*** and Its Ligands

PDGFR*α* is one of the two PDGF receptors (*α* and *β*) subunits [10, 11] found in higher vertebrates, which form homo- and heterodimers and interact with at least four PDGF ligands [12]. The *α* receptor, similarly to the *β* receptor, consists of extracellular, transmembrane, and intracellular portions. Ligand binding induces the dimerization of the receptors and autophosphorylation of specific tyrosine residues in their cytoplasmic domains. These phosphotyrosine residues serve as docking sites for adaptor proteins that initiate signal transduction.

PDGFRs are primarily regulated by their ligands which were originally described as a platelet-derived mitogen for fibroblasts and smooth muscle cells [13] released by activated platelets. Subsequently, PDGFs are found to be synthesized by a number of different cell types including immune cells and fibroblasts. Since then, four different PDGF chains have been characterised, namely, PDGF-A, PDGF-B, PDGF-C, and PDGF-D [14–17].* In vitro* studies have shown that PDGFs induce multiple cellular effects, for example, cell proliferation, transformation, migration, and survival [18]. They also play important roles during embryonic development and in many pathological processes, such as in wound healing, angiogenesis, atherosclerosis, and oncogenesis [18–20].

Both PDGFR*α* and PDGFR*β* engage several well-characterized signalling pathways, for example, mitogen-activated protein-kinase (Ras-MAPK), extracellular signal-regulated kinase, the phosphatidylinositol 3-kinase/Akt, and the phospholipase C-*γ* pathways, which are known to be involved in multiple cellular and developmental responses. Several additional signalling molecules are engaged by PDGFRs, including enzymes, adaptors, and transcription factors. For example, activation of the Src TK promotes c-myc transcription and mitogenic responses. The mitogenic activity of the PDGFs on fibroblasts and smooth muscle cells [13] is a well-known fact that led to the discovery of the growth factors.

## 3. *Pdgfr* ***α*** Progenitors in Development

**Figure 2:**
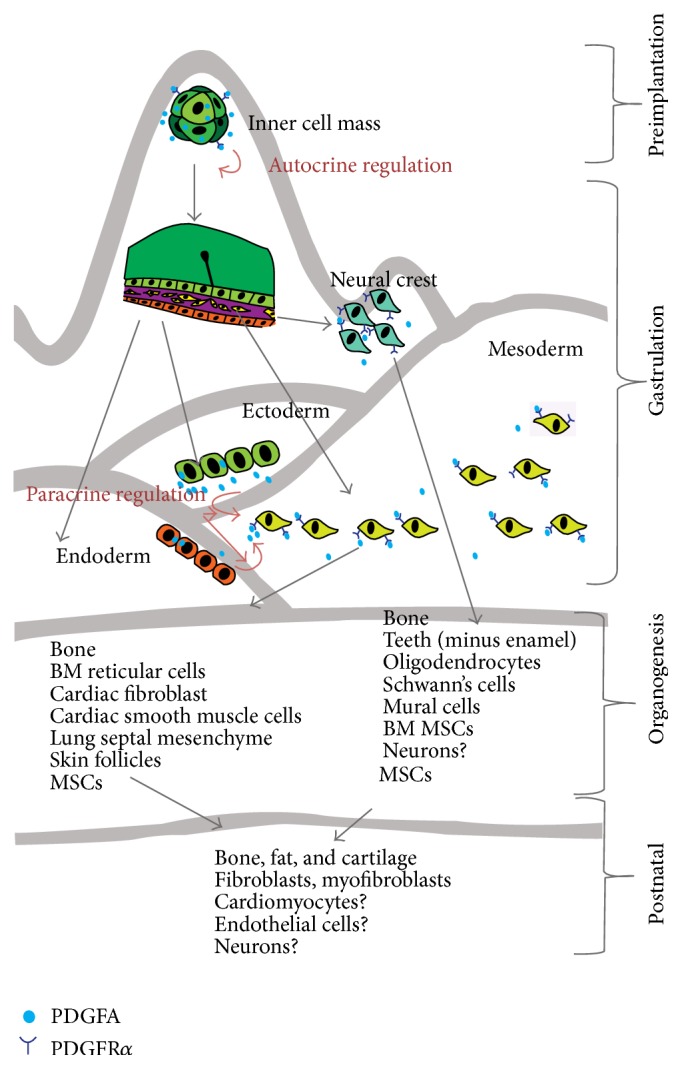
Schematic depiction of the differentiation hierarchies of PDGFR*α* ^+^ cells during embryogenesis and postnatal MSCs.

Intriguingly, PDGFR*α* expressions during the early stage of life to adulthood seem to follow Waddington's theory of marbles rolling down a hill, appearing at the different developmental stages, perhaps with increasingly restricted differentiation capacities, nevertheless, proceeding from totipotent to multipotent states and then restricting their ability to formation of reparative tissue (Figure 2).

Earliest expressions of the* Pdgfrα* mRNA are present at high levels in the fertilized egg, two-cell stage, and, at the blastocyst stage,* Pdgfrα* is expressed in the inner cell mass [10, 21]. The importance of the* Pdgfrα*-PDGFA signalling in the early embryos is not clearly demonstrated, as PDGFR*α* knockout in mammalians prior to gastrulation can be a redundant event. It is possible that the coexpression of its ligand PDGFA both at the mRNA and protein level [22, 23] may act in an autocrine manner to stimulate cell proliferation at a high rate that facilitates the rapid proliferation of the cell mass. Conversely, blocking the autocrine PDGF loop in early embryonic development is lethal in the Xenopus [24]. In malignancies, the autocrine relation may be evoked to increase tumour cell proliferation and Epithelial to Mesenchymal Transition (EMT) processes [25].

Despite an early expression in the embryos, the marker becomes largely confined to the mesoderm after the gastrulation [26]; for example,* Pdgfrα* is expressed in paraxial mesoderm, in most if not all mesodermal cells, and begins to be distributed throughout the embryonic mesenchyme [27]. This mesoderm derived mesenchymal population is shortly joined by another source of the* Pdgfrα* positive mesenchymal cells derived from the neural crest cell migration. Transient* Pdgfrα* expression has been observed in the most dorsal portion of the neural tube at E9 of mouse embryo, coinciding with neural tube closure, and was thought to represent the neural crest cells before their migration from the neural crest [28]. Collectively, these mesenchymal progenitors set out to expand the body's connective tissue components in coordination with parenchymal cell growth of the organs and interference with the* Pdgfrα* signalling is lethal to the embryos as most of the knockout mouse embryos die before E10 [29].

At this stage, the coexpression of receptor and ligand changes to an adjacent expression pattern such that PDGFR*α* becomes confined to the mesoderm and neural crest, whereas PDGFA is expressed in the adjacent ectoderm and endoderm [22, 26, 28]. The implication of the shifting pattern of expression from autocrine to paracrine PDGFA is unclear. A paracrine PDGFA might induce mesenchymal cell proliferation through an interaction between epithelium and mesenchyme, an interaction known to play an important role during induction and development of many organs. For example, in the development of limb buds, PDGFA is expressed in the surface ectoderm and muscles, while* Pdgfrα* is diffusely expressed in the mesenchyme, especially that surrounding the developing bones [26]. Similar interactions are also described in development of foregut, diaphragm, the pleural and pericardial membrane, and the urogenital, where the mesenchymal primordia express* Pdgfrα* [26, 30]. Presumably, adult MSC niche in the mature organ is carefully regulated via similar paracrine signalling to maintain homeostasis of the parenchymal cell functions.* PDGFRα* cells are present in stromal components of virtually all-adult organs, and misregulation of* Pdgfrα* is related to many pathogeneses. Examples include well-established links between mutation in the* Pdgfrα* and gastrointestinal stromal tumours [31]; age dependent impairment of PDGFR*α* in the pancreas and heart may lead to impairment of the *β*-cell insulin productivity [32] and cardiac function [33], respectively, suggesting a crucial regulatory role that MSCs play in maintaining health of the normal tissue.

Controlling of these interactions may provide strategies for disease treatment modalities. Indeed, PDGF signaling pathways have been used to augment cardiac functions after myocardial infarctions in rodents. Intramyocardial delivery of PDGF ligands has been reported to produce promising improvement of cardiac functions, mainly through augmentation of cardiac angiogenesis and reduction of myocardial damage [33–36], but the role of the ligands on the activation of endogenous PDGFR*α* ^+^ MSCs is not elucidated.

## 4. *Pdgfr* ***α*** Progenitors in Postnatal Organs

### 4.1. *Pdgfrα* Progenitors in Bone Marrow

MSCs were first isolated by Friedenstein et al. from BM [2] using their plastic adherent properties and have been extensively used in the clinical trials to treat disorders ranging from autoimmune diseases to cardiovascular pathologies, with varied results. Beneficial results are generally attributed to their paracrine-regulatory effects rather than an active engraftment to the diseased tissues and generation of parenchymal cell types. Recent studies show that the PDGFR*α* represent a population of MSCs when isolated from nonhaematopoietic compartment of the BM [37]. The CFU-F forming cells are positive for PDGFR*α* and SCA1 but negative for haematopoietic and vascular markers CD45 and CD31 and have augmented growth potential and robust trilineage (osteogenic, chondrogenic, and adipogenic) differentiation compared with standard culture-selected MSCs [4]. PDGFR*α* is shown to enrich STRO-1^+^ cells isolated from human BM to act as high-growth capacity MSCs. These cells also have the highest mean expression of the mRNA transcripts TWIST-1 and DERMO-1 and are capable of forming ectopic bone [38]. PDGFR*α* ^+^CD51^+^ may also enrich BM nestin^+^ cell population in the human foetal BM that has MSC and haematopoietic stem cells niche activities, as cultured human PDGFR*α* ^+^CD51^+^ nonadherent mesenspheres can significantly expand multipotent hematopoietic progenitors that engraft immunodeficient mice [39]. A neural crest origin is described for the BM MSCs [40], although debates exist whether mesoderm cells later replace this population. BM-derived PDGFR*α* ^+^ cells are shown to differentiate into ectodermal keratinocytes and mesenchymal dermal fibroblasts, particularly in the setting of skin grafts [41], and migrate to donor mouse skin graft and supplement collagen 7 in the basement membrane [42].

### 4.2. *Pdgfrα* Progenitors in the Heart

*Pdgfrα* is expressed at the venous pole in the mesocardium, the myocardium of the sinus venosus, the proepicardial organ, and the coelomic mesothelium in the E9.5 mouse embryos. At 14.5, the* Pdgfrα* cells disappear from the myocardium but are found in the epicardium covering the ventricles. The atrial ventricular cushions still express the receptor and some scattered cells are also found in the septum [29]. After E15.5, the* Pdgfrα* cells migrate into the myocardium and assume locations within the coronary arterial adventitia and are also found in the ascending aorta and more broadly within the cardiomyocyte interstitium close to or in contact with the basement membrane of microvessels [43]. Direct lineage tracing of the* Pdgfrα* cells in the heart has not been reported.* Wt1* lineage tracing studies have shown that* Pdgfrα* ^+^ cells coexpresses* Wt1*, perhaps indirectly suggesting that the* Wt1* ^+^ *Pdgfrα* ^+^ cells give rise to cardiac fibroblast and smooth muscles. Endothelium and cardiomyocyte derivations have been reported but the notion is not widely accepted as reviewed by Asli et al. [43]. Impairment of* Pdgfrα* in both mesoderm origin epicardially derived stem cell (EPDC) migration and the neural crest progenitors leads to myocardial thinning, epicardial blebbing, or septum defect and dying before birth [26, 29, 44]. The SCA-1^+^PDGFR*α* ^+^ cells isolated from the adult mouse hearts (Figures 1(a) and 1(b)) have a broad differentiation capacity* in vitro*. Limited* in vivo* differentiation is seen as only a few cardiomyocyte-like cells were found in the cultured and transplanted cells. An epicardial origin is defined for these cells [6]. More recent studies from Schneider's group confirmed Chong et al. reports that the SCA-1^+^PDGFR*α* ^+^ cells demarcate a population of clonogenic MSCs in the heart that originate from the epicardium [6]. Clonal progeny of single SCA-1^+^PDGFR*α* ^+^ (defined by Hoechst 33342 dye-efflux in fluorescence activated cell sorting system) cells shows cardiomyocyte, endothelial, and smooth muscle lineage potential after cardiac grafting, augmenting cardiac function, although durable engraftment is rare [45]. Reactivation of EPDC population is reported to participate in the repair of the adult myocardium largely via generation of fibroblast population, indicating potential therapeutic target for endogenous MSC activation, although cardiomyogenic capacity is debatable [43, 46].

### 4.3. *Pdgfrα* Progenitors in Neuronal System

The role of* Pdgfrα* in the central nervous system (CNS) is not completely clear since there is still a doubt as to whether the neuronal cells express* Pdgfrα*. It appears that the* Pdgfrα* mRNA is not present until after E12.5 in the mouse CNS. A spotted* Pdgfrα* signal starts from the ventral area of the central canal of the spinal cord and spreads throughout the whole spinal cord and restricted areas in the brain. These* Pdgfrα*-expressing cells have been identified as Oligodendrocyte Progenitors (OPs) [47–49]. However, others have shown that* Pdgfrα* is also expressed in some neurons, for example, in the neurons of the retina, Purkinje cells of the cerebellum, and some ventricular zone cells of the brain [50].* Pdgfrα* have been reported to colocalize with NG2-expressing glia and are distributed throughout the adult CNS. They are descended from OPs in the perinatal CNS. By following the fate of adult OPs in* Pdgfrα*-*cre*ER(T2)/*Rosa26-*YFP double-transgenic mice, Richardson's group has found that they generate many myelinating oligodendrocytes in corpus callosum during adulthood [51]. Labelled* Pdgfrα* ^+^ cells have been reported to generate neurons and oligodendrocytes* in vivo*. However conditional ablation of the receptor in the postnatal stem cells (a subpopulation of B cells at the subventricular zone) does not block neurogenesis; therefore the authors concluded that signalling occurs early in the adult stem cell lineage and may help regulate the balance between oligodendrocyte and neuron production [52]. Sorted foetal human forebrain cells for PDGFR*α* are differentially expressed by OPs. When transplanted these cells were highly migratory and robustly myelinated the hypomyelinated shiverer mouse brain more rapidly, confirming the myelinogenic progenitor cells [53]. In addition,* Pdgf-A* knockout mice show a dramatically reduced number of OPs in the CNS and a reduced number of oligodendrocytes together with a dysmyelinating phenotype. Also, the PDGFR*α* antibody APA injection into neonatal mice resulted in reduced oligodendrocytes population in the retina [54]. Conversely, overexpression of* PdgfA* can stimulate OP reentry to cell cycle and hyperproliferate [55].

### 4.4. *Pdgfrα* Progenitors Cells in Skeletal Muscle and Adipose Tissues

*Pdgfrα* cells are found in the interstitium of the human skeletal muscle (Figure 1(c)) and are distinct from the satellite cells. When isolated and subjected to adipogenic differentiation, these cells displayed not only adipogenic but also fibrogenic potentials while CD56^+^ satellite cells differentiate into myocytes under the same conditions [8]. Human skeletal muscle* Pdgfrα* cells are isolated to induce formation of bone-like tissue* in vivo* by coimplantation of the cells seeded in a hydroxyapatite block [56]. However, it is not clear whether* de novo *bone progenitors in the BM are* Pdgfrα* positive.

*PdgfRα-Cre* lineage tracing in postnatal mice has shown that white adipocytes are derived from these progenitors. When the sorted* Pdgfrα* cells were injected subcutaneously over the sternum of wild-type mice, these cells resulted in formation of mature adipocytes in this region where adipose tissue does not normally form. These cells are coexpressed with CD24 and negative for haematopoietic and endothelial markers [57].

### 4.5. *Pdgfrα* Progenitors in Head and Epithelial Tissues

After gastrulation,* Pdgfrα* is clearly expressed in the mesenchyme of branchial arches of E9-11.5 mouse embryos, a type of tissue which is of ectoderm-derived neural crest cell origin [30]. The branchial arch mesenchyme later contributes to the craniofacial neural crest cells and gives rise to facial bone primordia, teeth, dermis, papillae of skin follicles, and the connective tissue surrounding the eye.

PDGFR*α* cells are also found in the subepithelial layer of the mouse and human colon. The cells are distinct from SMA^+^ myofibroblasts of the colon and PCR analysis on the isolated PDGFR*α* revealed genes of Toll-like receptor and 5-Hydroxytryptamine, indicating that these cells may participate in innate immune responsive and neural transduction activities of the colon [58].

Clonal thymic mesenchymal cell lines were found to express SCA1 and PDGFR*α* similar to MSCs that can be maintained in culture. Sorted cells contribute to thymic architecture when reaggregated with foetal stroma and transplanted under the kidney capsule of nude mice suggesting their contributions to maintenance of functional thymic microenvironments [59].

In the adult lungs, around 29% of the mesenchymal population expresses* Pdgfrα* (Figure 1(d)), which can recapitulate the stem cell niche and support growth and differentiation of the alveolospheres generated from type 2 alveolar stem cells. The lung* Pdgfrα* cells express LipoTOX [60], a marker of the so-called lipofibroblasts.

*Pdgfrα* is expressed in both the upper and lower dermis at all stages of development. Recent studies by Driskell et al. have elegantly demonstrated the contribution of* Pdgfrα* ^+^ cells to the dermis architecture by labelling these cells using a tamoxifen inducible Cre method at pre-E16.5 and adult mice and inducing dermis wound at an adult age [61]. In pre-E16.5 embryos, the labelled cells contributed to the mesenchymal components of hair follicle, glands, and papillae of the skin. At E16.5, the contribution becomes more restricted to adipocytes and hypodermis. In adults, only scar-forming fibroblasts were seen to form from the transplanted cells [61], demonstrating heterogeneity of the PDGFR*α* ^+^ cells during development and lineage specification restrictions that occur during aging.

## 5. Summary

Taken together, PDGFR*α* signalling is important in mesenchymal cells and connective tissue growth during development. They predominantly contribute to the connective tissue types such as bone, teeth, adipose, and stromal components of the organs and provide architectural supportive niche for parenchymal cells of specific organs. There is less evidence supporting parenchymal cell type differentiation of the PDGFR*α* cells after organogenesis. Isolated MSCs seem to have tendencies to primarily give rise to fibrogenic and adipogenic cells. PDGFR*α* ^+^ cells locate broadly in the perivascular niche of the adult tissue, may play supportive role in tissue homeostasis, and provide paracrine as well as cellular components to support repair of the host organ/tissue, with subfraction of the cells possessing MCS-like characteristics. Increasing lines of evidence point to the direction that PDGFR*α* may be the most promising marker for studying the roles of mesenchymal stem/progenitor cells, their anatomical location, and contribution to the tissue repair* in vivo*. SCA-1 positivity is frequently associated with isolation of the PDGFR*α* ^+^ cells in the adult murine tissues. PDGFR*α* ^+^ cells are demarcated from the endothelial and haematopoietic populations of the selected tissues. Some other coexpressing markers have been reported such as CD51^+^ in the BM and Hoechst 33342 dye-efflux characteristics in the cardiac tissues as well as NG2 positivity in neural progenitors. Using PDGFR*α* ^+^ as a marker provides one step higher resolution in dissecting the stromal population of the organ/tissue in the process of harnessing the innate ability of these cells, as well as facilitating the isolation, enrichment, differentiation, and functional studies of the MSCs without contamination from the broader fibroblast family cell types. Targeting PDGFR*α* ^+^ mesenchymal progenitors might open new opportunities for designing therapeutic strategies for disease control and tissue regeneration.
